# Centralized clinical research operations reporting for a multi-network, multi-study research program: The NHLBI COVID-19 CONNECTS experience

**DOI:** 10.1017/cts.2025.10189

**Published:** 2025-11-11

**Authors:** Kayla Nowak, Sean Hanlon, Jeanette Auman, Heather Meier, Katherine Asman, Tracy Nolen

**Affiliations:** RTI International, Research Triangle Park, NC, USA

**Keywords:** Clinical research coordination, central reporting, web portals, network of networks, COVID-19

## Abstract

The Collaborating Network of Networks for Evaluating COVID-19 and Therapeutic Strategies (CONNECTS) was a novel network of networks created in response to the COVID-19 pandemic. This program brought together a large matrix of clinical research networks to swiftly design and/or implement concurrent clinical studies. Successful coordination of this large-scale collaboration required innovative solutions for timely and transparent centralized operations reporting. As the Administrative Coordinating Center (ACC) for CONNECTS, RTI International developed and maintained a web-based infrastructure that served as the central communication and reporting hub. This single-platform approach provided a robust collection of key topics to support daily operational oversight (e.g., enrollment and retention, site coverage, study milestones, financial tracking). Underlying data acquisition, harmonization, and portal reporting methods aimed to address nuances of the network of networks (e.g., disparate data sources, diverse user needs) while providing the necessary speed and agility to combat the COVID-19 pandemic. The resulting system was well received, readily adapted to changes, and successfully supported three years of early COVID-19 research. Although the CONNECTS central reporting methods arose from necessity during an urgent and dynamic public health emergency, they are a model for efficient and effective centralized operational reporting for any large-scale public health research effort.

## Introduction

The Coronavirus Disease 2019 (COVID-19) rapidly reached pandemic status and presented a worldwide public health emergency within a few months of outbreak. The urgency to find treatments for COVID-19 challenged the U.S. clinical research infrastructure. Nothing was known about the disease early in the pandemic, and new information was desperately needed to combat the virus and save lives. Thus, there was an unprecedented call for swift research collaboration at the national level.

**Figure 1.**
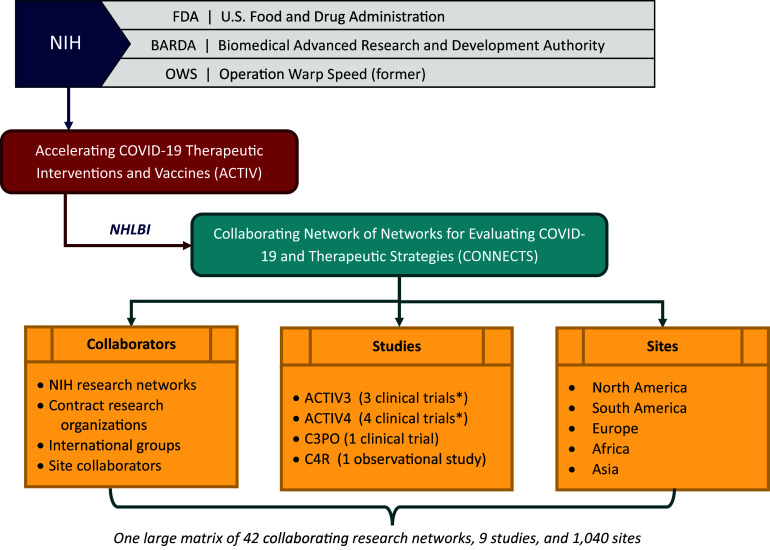
CONNECTS structure and partnerships. C3PO = Clinical Trial of COVID-19 Convalescent Plasma in Outpatients; C4R = Collaborative Cohort of Cohorts for COVID-19 Research; NIH = National Institutes of Health; NHLBI = National Heart, Lung, and Blood Institute. * Includes adaptive platform trials with multiple, added treatment arms.

In spring of 2020, the National Institutes of Health (NIH) formed the Accelerating COVID-19 Therapeutic Interventions and Vaccines (ACTIV) initiative, which brought together researchers and resources from established research networks to rapidly develop diagnostics, vaccines, and therapeutics for COVID-19 [1]. As part of this initiative, the National Heart, Lung, and Blood Institute (NHLBI) formed the Collaborating Network of Networks for Evaluating COVID-19 and Therapeutic Strategies (CONNECTS) with the goal to rapidly design and implement adaptive master protocols to evaluate host-directed COVID-19 therapeutics [2]. NHLBI instituted a “network of networks” approach in which researchers from multiple existing and established NHLBI-funded clinical research networks came together under one organizational umbrella to form seamless and strategic collaboration across a matrix of concurrent studies and co-enrolling sites (Figure 1). This effort ultimately brought together over 68,700 participants (11,000 clinical and 57,700 observational) across 1,040 sites, 42 research networks, and 9 studies (8 trials and 1 large observational study).

Successful coordination of a program of this scale, urgency, and complexity required timely and transparent centralized communication. Government oversight agencies and collaborators at the site, network, study, and program levels all needed daily updates to monitor progress and swiftly identify operational challenges. However, relevant information varied by user, source data were disparate across networks/studies, and reporting needs were expected to change as COVID-19 research evolved. To address these necessities and challenges, RTI International, serving as the CONNECTS Administrative Coordinating Center (ACC), developed and maintained a web-based cloud infrastructure for centralized CONNECTS operational reporting. The infrastructure employed a strategic and flexible central reporting framework that prioritized interactive dashboard displays and leveraged multi-cloud technology. This design was supported by an innovative data processing and reporting workflow to acquire, harmonize, and report operations data each day. The methods, successes, and limitations of this central reporting approach are described herein to inform future collaborative central reporting efforts.

## Methods

Traditional, often time-consuming, design and development processes were untenable due to unprecedented circumstances of the early pandemic. Instead, the ACC established a core reporting framework and data workflow to support rapid launch and future updates. This condensed planning process was guided by key design principles from public health surveillance systems and performance management systems: collaborators were engaged early in the design process; simple designs were prioritized for easier interpretation and adaptation; timeliness, flexibility, and stability were considered when selecting methods for implementation; and usefulness of the reporting features was at the forefront of design conversations [3–5].

### Central reporting framework

A web-based cloud infrastructure was selected as the central platform for CONNECTS program communications. This included a private portal for program-wide operations oversight and monitoring, plus a public site with general information about CONNECTS to encourage research transparency and scientific community engagement.

The private portal had restricted, role-based user access and included operational data reports for all studies in the program. Early in the design process, the ACC worked closely with NHLBI to identify primary domains of operational oversight: participant enrollment and retention, site activation and geographic coverage, study design and implementation milestones, and financial tracking. Domains served as the underlying reporting structure to provide a solid foundation for both immediate reporting needs and future expansion. As reporting needs changed over the pandemic, additional features or display types could be added to a domain without compromising the original architecture. Initial report designs prioritized dashboard-type displays to quickly and intuitively disseminate large amounts of information. Real-time interactive filtering for network, study, and/or site were included in most displays to accommodate the diverse user groups, varying research priorities, and different operational questions. Clinical and observational studies had separate filtering menus to simplify “select all” viewing (e.g., select all clinical vs. select all observational); users could also select both study types to view all program studies.

To ensure scalability and reliability, the CONNECTS web infrastructure utilized a multi-cloud approach that leveraged services provided by Microsoft Azure and Amazon Web Service (AWS). Private portal security required user authentication (i.e., username and password) and was engineered using contemporary and established technologies and techniques (e.g., relational databases, Microsoft’s .NET Framework, web application frameworks, microservices architecture). Within the private portal, user roles determined content and functionality access. All users were assigned a role by CONNECTS project management at onboarding, and role types were tailored for various operational reporting needs (e.g., committee members, financial oversight, site start-up, and enrollment monitoring).

### Data processing workflow

**Figure 2.**
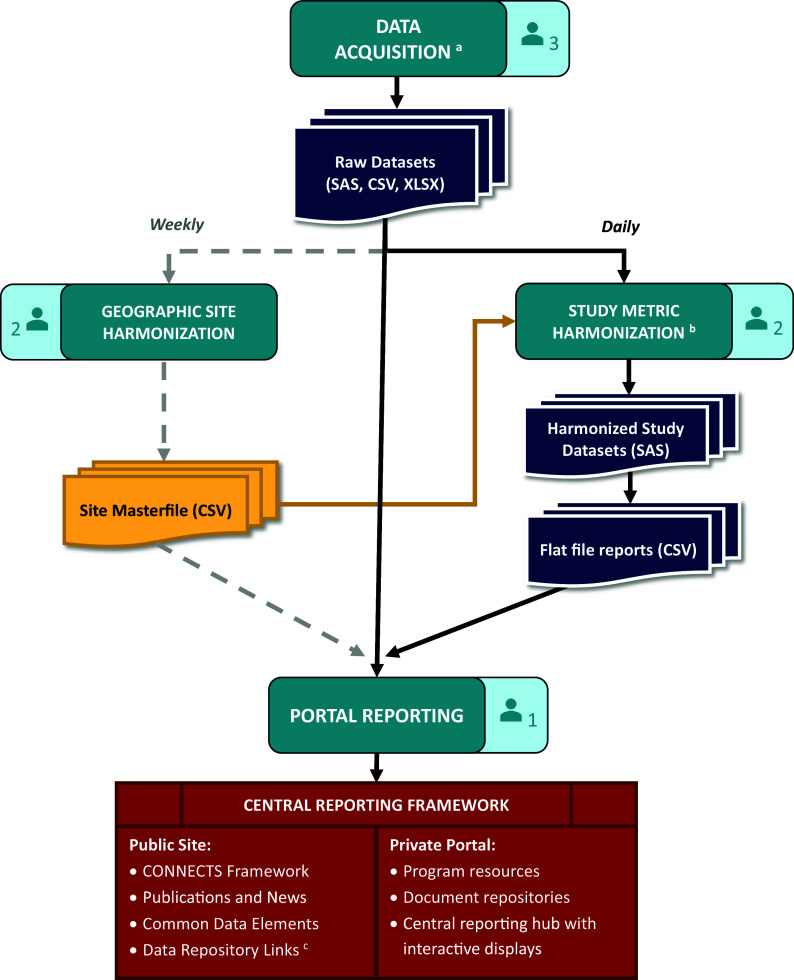
CONNECTS data processing and reporting workflow. ^a^Data acquisition included site, center, and/or participant-level data sourced from the DCCs, as well as internal data management systems (e.g., milestone progress, financial snapshots) and external data sources (e.g., COVID-19 infection data from the Johns Hopkins University’s Coronavirus Resource Center [8]). ^b^Harmonized study data were limited to recruitment, retention, and demographics. ^c^CONNECTS study data were publicly available through NHLBI BioData Catalyst [7]. *Note*: Each of the four workflow stages (data acquisition, study metric harmonization, geographic site harmonization, and portal reporting) includes an icon indicating the size of the team. Three statistical programmers were divided across the two harmonization steps: one for sites, one for study metrics, and one supporting both efforts.

CONNECTS central reporting was achieved through a data processing and reporting workflow that comprised data acquisition, study metric harmonization, geographic site harmonization, and ultimately, portal reporting. This workflow (Figure 2) spanned several teams and ran daily to ensure that the CONNECTS portal included the most current data available. The acquisition team included two software developers and one clinical data manager, the harmonization team included three statistical programmers, and portal reporting was accomplished by one software architect/engineer.

#### Data acquisition

Operational study data for portal reporting included site identification and start-up, participant enrollment, demographics (e.g., age, sex, race, ethnicity), and study retention. For each study, these data were sourced from four separate Data Coordinating Centers (DCCs) which required extensive acquisition procedures. Since CONNECTS was built on existing networks, each participating study’s DCC employed their own study management and electronic data capture systems, resulting in disparate data that required harmonization prior to portal ingestion. Additionally, study teams were required to follow their institutional regulatory and data governance policies, which necessitated data transfer via different pathways.

The ACC needed to be amenable and adaptable to the varying formats and pathways and met with study teams early in the process to establish data acquisition solutions tailored to each DCC’s requirements. This resulted in accessing the data via a variety of methods (e.g., secure file transfer protocol [FTP] accounts, application programming interfaces [APIs], web scraping) and acquiring files in varying formats (e.g., HyperText Markup Language [HTML] tables, comma-separated values [CSV] files, Excel workbooks, and SAS files). For efficiency and reliability, custom scripts were used to automate extraction and convert files into more easily digested formats. Elements of the script ran daily, weekly, or monthly based on the frequency of source data updates. Other types of data for portal reporting were directly ingested daily from internal (e.g., RTI’s finance and SharePoint systems) and external sources (e.g., PubMed, COVID-19 infection data).

#### Study metric harmonization

Data acquired from the DCCs required content and structural harmonization for central reporting. Core reporting metrics (e.g., site status, participant demographics, enrollment) were captured with different variable names and response options across studies. Also, some sources were participant-level records, others were site-level summaries, and some summaries were weekly snapshots of content that varied week to week. Due to the nature and timing of CONNECTS, DCCs were not required to standardize or harmonize operational data for central reporting activities; however, DCCs were responsible for harmonizing complete study data to the COVID-19 common data elements for public data sharing [6,7]. Harmonization efforts for portal reporting were completed by the ACC.

Content harmonization aimed to maximize data inclusion from disparate sources while preserving meaningful metrics for CONNECTS oversight. This balance often resulted in reporting metrics at the lowest common value across existing studies. For example, CONNECTS reported broad age categories (<18, 18 to 30, 31 to 64, or >64) to support sources with varying granularity of age data. Structural harmonization utilized a flat file approach: harmonized data were summarized to the lowest common level of central reporting (site, within network, within study), and one output file was produced per study, per domain. This optimized portal integration and operability, while accommodating pre-summarized and participant-level data sources.

Content and structure harmonization were accomplished through a series of study-specific programs that automatically launched as a daily batch script. Each program applied custom code to harmonize source data and then called a set of standardized reporting macros (one per domain) to create the flat file outputs. Automated quality checks were included throughout, and the last step of the series generated a “success” (no errors) or “failure” (one or more errors; issues listed) email to serve as the final, daily quality check before new data were released to the portal. Simple errors (e.g., script failures, new supplemental data fields) were quickly addressed by the ACC prior to the daily portal refresh. Larger issues (e.g., full data restructuring, changes to core data fields) required discussion with the source DCC, initiated by the acquisition team lead. In such cases, the daily portal refresh used the last error-free version of flat file reports for the affected study/studies.

#### Geographic site harmonization

Individual study sites also required harmonization for central reporting. Under the network of networks design with concurrent trials, a medical site (e.g., hospital, university, clinic) could originate from multiple research networks and participate in multiple studies across the program. Tracking program-wide site locations identified enrollment competition risks across studies from co-located clinical sites and provided established recruitment pathways for new studies. Additionally, the geographic aspect supported strategic recruitment efforts based on the spread of COVID-19. For these reasons, site-level reporting was identified early in the design process as an essential component for CONNECTS oversight, and harmonized sites were selected as the lowest common level of portal reporting.

Study site identification data (i.e., site name and street address) were captured during data ingestion; however, different data sources could report a different name and/or address for the same physical location, and some sources were unable to provide complete address information. Additionally, new sites joined CONNECTS each week as research continued to expand. To accommodate disparate and dynamic site data, a geographic harmonization step was added to the data processing workflow. This step identified and harmonized new study sites once a week to minimize disruptions to the portal while still ensuring data freshness.

Geographic harmonization utilized Google Maps API to locate and geocode new study sites based on supplied information. Hierarchical clustering with cut-tree methods (R hclust and cuttree functions) were then applied to the geospatial data frame to group geographically similar sites. A strict 320-m cut criteria was used to represent approximately 1.5 city blocks, where geographically similar sites had a greater likelihood of being the same physical site. These steps were automated for efficiency and served as a first pass at harmonization. Automated findings were manually reviewed and updated as needed before final harmonization assignments were committed to tracking files.

#### Portal reporting

Acquired (raw) and harmonized study data were ingested by the CONNECTS web interface using extendible and flexible methods. Information onboarding logic was componentized to support re-use, expansion, and specificity when warranted. Harmonized study data were integrated every morning following the automated “success” email, and directly acquired data sources were automatically ingested by the infrastructure daily. Geographic harmonization data updates were pushed weekly during off-hours to minimize disruptions since site was a key structural element for the interactive displays. Data transfer dates for each study were published on the private portal to convey data freshness.

## Results

The CONNECTS web presence (https://nhlbi-connects.org/) was first released on August 5, 2020, just 2 months after the ACC was established. The private portal was accessible to a diverse group of 635 users, including NHLBI partners, external collaborators, oversight entities, network researchers, study principal investigators, CONNECTS leadership, and ACC staff. By the end of CONNECTS study enrollment (August 2023), public and private sites had over 92,000 total page views.

Oversight groups and collaborators relied on the portal for daily monitoring, but individual study teams also used the portal to support their own monitoring needs. Notably, the site start-up reports were often highlighted as an essential tool for managing the various steps for rapid site activation. User feedback from ACC-led training sessions and technical support emails suggested that the portal was well received, and the only revision requests were for additional reporting elements/features in response to changing research questions. Daily meeting between the sponsor and ACC assessed and prioritized infrastructure updates based on user feedback and advancements in COVID-19 research.

### Central reporting framework

**Figure 3.**
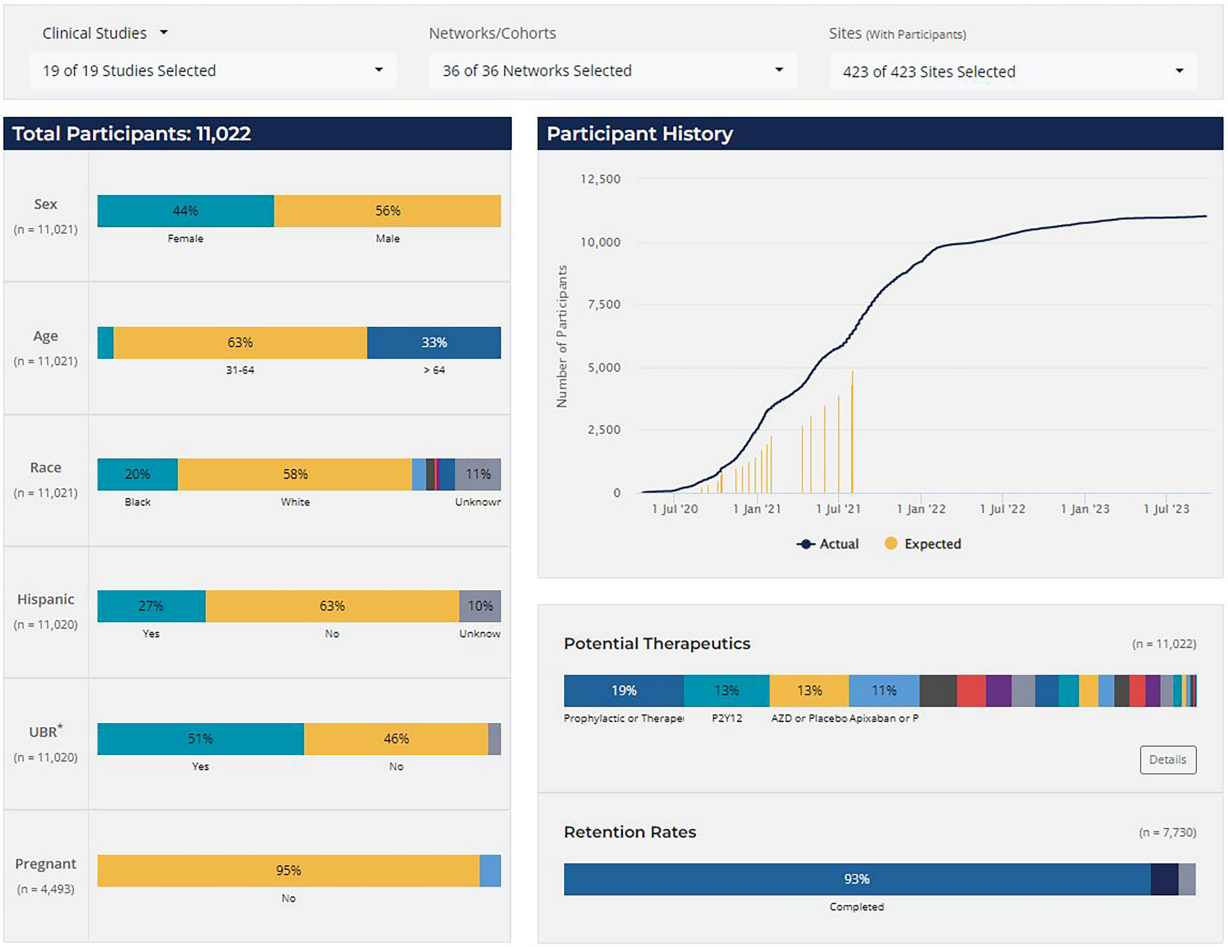
CONNECTS study accrual and retention report, clinical studies. *UBR = underrepresented in biomedical research, defined as individuals that identify as either non-white or Hispanic/Latino. *Note*: The accrual and retention reporting snapshot was taken in July 2024. The selected display view includes combined data from various phases of the clinical studies (ACTIV4a, ACTIV4b, ACTIV4c, ACTIV4 host tissue, C3PO, ACTIV3, ACTIV3b, and ACTIV3c); data from the C4R observational research program are not shown. Expected enrollment data were not available for all studies or all study phases.

Study Accrual and Retention (Figure 3) was the most frequented report type, used for daily enrollment monitoring. The single-glance dashboard display provided a quick overview of enrollment trends, demographic representation, therapeutics coverage, and retention rates. Interactive features allowed users to filter the display by study, network, and/or site for customized monitoring needs. Based on user feedback, two more interactive displays were added to this report over time: a second dashboard dedicated to site activation metrics and a tabular (exportable) version with details for both enrollment and site startup.

**Figure 4.**
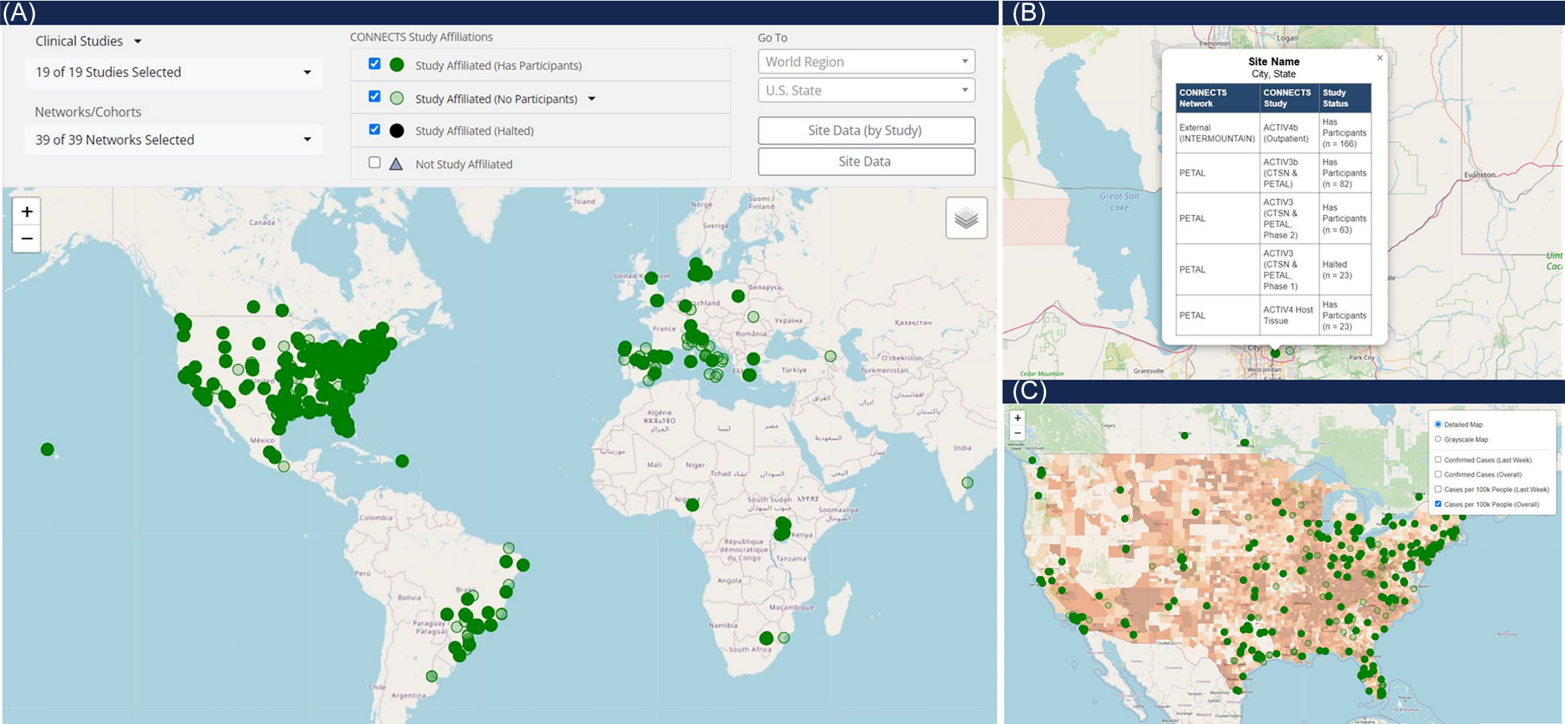
CONNECTS network map. Panel A is a full view of the CONNECTS network map reporting element, showing all study affiliated clinical sites as of July 2024. Panel B shows the pop-out feature from selecting a site marker. The pop-out lists the harmonized site name (masked), city and state (masked), all networks and studies present at the location, and the enrollment status of each network and study at the site. Panel C shows an alternate view of the map, focused on the continental U.S., and with the heatmap overlay option selected for total COVID-19 cases per 100k people. COVID-19 case data were sourced from Johns Hopkins University’s Coronavirus Resource Center [8].

The map of harmonized CONNECTS sites (Figure 4, panel A) was the second most frequented report type, primarily used by the ACC and program collaborators to monitor CONNECTS’ global presence and prospectively identify new study sites. This interactive global map included markers for all CONNECTS-affiliated sites, and selecting a marker opened a pop-out box listing all study enrollment statuses at the location (Figure 4, panel B). The map was filterable by study and/or network and fully scalable to accommodate large- and small-area viewing. Additionally, several heatmap overlay options were available to depict COVID-19 case density in the United States, sourced from Johns Hopkins University’s Coronavirus Resource Center (Figure 4, panel C) [8]. The combined interactive features in this single display created a powerful and informative tool for targeted oversight, rapid projections, and mobilizing research in areas of high infection rates. The underlying data for this display were later made available as an exportable data frame to facilitate user-driven ad hoc reporting.

Additional reports on the private portal included study milestone trackers and financial reports for specialized NHLBI users. These reports used a combination of graphic summaries and tabular listings, which were interlinked and interactive to filters. The private portal also housed document repositories for various committees and boards who advised on program activities in response to findings from the scientific community at large.

### Data processing workflow

Automated procedures and quality checks ensured fast and reliable daily processing for portal data refresh by 9am Eastern Time most days. Even during peak CONNECTS operations (2021–2022), the daily workflow required less than 10 minutes of hands-on interaction. Weekly geographic site harmonization methods were equally efficient. A typical week of approximately 100 new study-sites required minutes to auto-harmonize and a few hours to manually review. This was a dramatic improvement from early attempts which took 20–50 hours to manually process a batch of 100 new sites.

## Discussion

During the critical early years of the COVID-19 pandemic, CONNECTS rapidly designed, implemented, and prioritized therapies for COVID-19 research with national-level collaboration, reflecting the key elements for success cited by Drs. Lane and Fauci in July 2020 [9]. Although there were many factors that contributed to the unprecedented speed in which CONNECTS studies progressed from start-up to result publication [2], the speed in which studies enrolled and reported results were a testament to the strengths of CONNECTS’ central reporting approach.

### Advantages

Having one central platform for all of CONNECTS’ operations reporting provided a single, comprehensive data source for program-wide oversight. This ensured that all collaborators and decision-makers had access to the same, current snapshot of information. Furthermore, by consolidating operational data from many studies, networks, and sites, the portal data provided a more robust set of information than what could have been accomplished from any one group alone. For example, monitoring concurrent study enrollment efforts at a single site was essential for CONNECTS success; however, this task would not have been possible without harmonized sites and reports available at the site, network, and study levels.

The strategic design of the central reporting framework ensured that the single platform approach met the needs of our diverse user group and successfully streamlined program communications. Simple, graphic reports were visually intuitive and effectively conveyed key operational metrics in an easy-to-digest format, yet details were available where needed through interactive features and data downloads. User roles and role-based access further improved portal user experience by supplying each user with direct access to relevant content.

Early launch of the CONNECTS web presence quickly established the portal as a reliable and accessible resource, while sustained production and expansion reinforced its value over time. Intentionally flexible design choices meant that a perfect system was not needed for the initial launch, which allowed for rapid production of both the web infrastructure and data processing workflow. A multi-cloud approach ensured that the infrastructure itself was reliable and scalable, while simple organization choices (e.g., study-centric acquisition, domain-centric reporting, core filter options) paired with repeatable and extendable programming methods proved effective and agile. Both the processing workflow and the web infrastructure readily accommodated new studies or arms within platform trials, new report types, and new reporting elements. The resulting system was highly efficient and successfully supported three years of daily updates with minimal user disruptions.

### Limitations

The CONNECTS approach was designed for administrative coordinating objectives. As such, the reported content, though diverse, was restricted to operational oversight and monitoring functions. While nearly all choices would translate well to other content and objectives, the benefits of ACC-led data harmonization would diminish with the addition of more complex or non-operational reporting domains (e.g., intervention adherence data, safety monitoring, biospecimen tracking, assessments/outcomes). These topics tend to be more study-specific, so DCC-led harmonization would likely be most efficient. Future large collaborations that are formed before study development and/or led by a central DCC could also take advantage of direct electronic health record queries or standardize case report forms. Data standardization greatly reduces the harmonization burden for both central reporting and public data sharing.

For CONNECTS, shifting the onus of acquiring and harmonizing operational study data to the ACC generated crucial time savings during the early months of the pandemic. By forgoing prior data standardization or a central electronic data capture system, networks were instead able to focus on rapid start-up, study enrollment, and participant care. Automated processes and the strategic balance of custom and standardized procedures in the data processing workflow made this shift not only possible but also efficient.

### Lessons learned

Although the CONNECTS infrastructure was adaptable to shifting oversight needs, the ACC underestimated the scope and demand for ad hoc reporting. The state of COVID-19 research in 2020 and early 2021 was so dynamic that operational priorities changed week to week. As a result, new and urgent (i.e., 48-hour fulfillment) reporting needs were addressed by the ACC as ad hoc reports. Most requests pertained to site start-up (e.g., quantify delays in contract execution, summarize site coverage in a specific state, identify established sites with the bandwidth to join new studies). Although each request had a unique objective, frequent requests for related topics exposed underlying knowledge gaps which were ultimately used to inform portal updates (e.g., new metrics, displays, or features). In response to the overall high demand for custom reports, most portal domains were later equipped with exportable data frames to facilitate on-demand, user-driven data explorations. Future endeavors would benefit from including data exports and a tool/tracker for ad hoc requests in the initial design.

Additionally, despite best attempts to build a flexible workflow and system, some patches and retrofits were still required. For example, streamlined portal ingestion depended on a consistent flat file structure for each domain. As new metrics were added to a domain, files from closed studies or closed study phases required retrofitting to match the new structure (even if the new metric(s) were null). This meant that harmonization programs from closed studies still required some level of upkeep months (or years) after the study’s final enrollment. Although this effort was manageable for CONNECTS, it was complicated by the same choices that streamlined daily processing. For example, harmonization programs maximized computational efficiency by using temporary datasets across reporting macros. However, this created programmatic dependencies, so updating one domain often required updates to all domains. Additionally, programmatic quality checks monitored for null/unexpected fields and outdated data snapshots. For retrofitting runs, these quality checks needed to be temporarily disabled. Future designs may wish to prioritize programming methods with flexible quality checks or fewer dependencies.

## Conclusion

Innovations and efficiencies of the CONNECTS’ cloud-based web infrastructure and development workflow supported the timely, transparent, and detailed operational reporting necessary for a novel “network of networks” design of concurrent trials led by multiple coordinating centers. The CONNECTS experience was unique due to the rapid onset and spread of COVID-19, which forced a “learn while doing” mentality; however, the resulting methods provide an exemplary framework of centralized reporting for any large-scale administrative or data coordinating effort. Similar web-based portal reporting methods have since been implemented by RTI for additional NIH COVID-19 research (e.g., Researching COVID to Enhance Recovery [RECOVER] observational studies and clinical trials) [10,11].
